# A dataset of remote-sensed Forel-Ule Index for global inland waters during 2000–2018

**DOI:** 10.1038/s41597-021-00807-z

**Published:** 2021-01-25

**Authors:** Shenglei Wang, Junsheng Li, Wenzhi Zhang, Chang Cao, Fangfang Zhang, Qian Shen, Xianfeng Zhang, Bing Zhang

**Affiliations:** 1 School of Earth and Space Sciences, Peking University, Beijing, China; 2 Key Laboratory of Digital Earth Science, Aerospace Information Research Institute, Chinese Academy of Sciences, Beijing, China; 3 University of Chinese Academy of Sciences, Beijing, China; 4 School of Earth Sciences and Resources, China University of Geoscience (Beijing), Beijing, China

**Keywords:** Limnology, Physical oceanography

## Abstract

Water colour is the result of its constituents and their interactions with solar irradiance; this forms the basis for water quality monitoring using optical remote sensing data. The Forel-Ule Index (FUI) is a useful comprehensive indicator to show the water colour variability and water quality change in both inland waters and oceans. In recent decades, lakes around the world have experienced dramatic changes in water quality under pressure from both climate change and anthropogenic activities. However, acquiring consistent water colour products for global lakes has been a challenge. In this paper we present the first time series FUI dataset for large global lakes from 2000–2018 based on MODIS observations. This dataset provides significant information on spatial and temporal changes of water colour for global large lakes during the past 19 years. It will be valuable to studies in search of the drivers of global and regional lake colour change, and the interaction mechanisms between water colour, hydrological factors, climate change, and anthropogenic activities.

## Abstract (summary)

**Table:** 

Measurement(s)	color
Technology Type(s)	satellite imaging
Factor Type(s)	temporal interval
Sample Characteristic - Environment	water body • lake
Sample Characteristic - Location	global

Machine-accessible metadata file describing the reported data: 10.6084/m9.figshare.13257218

## Background & Summary

Lakes are widely recognised as sentinels of environmental change, representing ecosystems that are particularly vulnerable to anthropogenic disturbance and climatic variability^1^. They play a crucial role in the global hydrological cycle, supporting extensive services such as water supply, hydropower generation, flood mitigation, fisheries, and biodiversity^2–4^. Deriving water quality information for lakes over large areas and long-time scales is of considerable value in exploring how lakes change and respond to environmental changes. Satellite remote sensing can potentially provide objective, broad scope, high frequency, and continuous measurements of inland water quality by capturing water colour information^5^. However, challenges brought about by the optical complexity of inland waters and overlying atmosphere, and interference due to adjacency effects have hindered the development of valid Earth observation (EO) approaches for water quality monitoring in inland waters compared with the ocean applications. As a result, few water quality EO products are available for inland waters at global and regional scales.

Water colour itself is recognised by the Global Climate Observing System as a key essential climate variable for lakes as it is directly related to variations in water constituents. Water colour is one of the oldest water observation data with records for global water bodies stretching back over a century. Water colour observations are based on the fact that clear water appears blue while turbid water turns green and/or yellow with increased levels of suspended sediment, phytoplankton, and coloured dissolved organic matter. It is traditionally measured using the Forel-Ule water colour scale, which divides water into 21 colour classes from dark blue to yellowish-brown. Recently remote sensing data have been applied to derive the Forel-Ule Index (FUI) of water using remote sensing reflectance (*R*_*rs*_) in the visible domain^6–8^. Studies have shown that FUI derived from *R*_*rs*_ has relatively low uncertainty due to its tolerance of aerosol perturbations, variable observational conditions, and good transferability across different sensors^7,9,10^. As water colour is the outcome of interactions between sunlight and the absorption and scattering of water constituents, changes in water optically active constituents can be described by variations in FUI^6,11^. The relationships between FUI and water quality parameters (e.g. chlorophyll-a (Chl-a) and total suspended matter (TSM), coloured dissolved organic matter (CDOM), turbidity, and water clarity) have been previously explored and documented^6,7,12,13^. While FUI yields more information on Chl-a for open oceans^11,12^, it is well-correlated with water clarity for coastal and inland waters according to the recent studies^12,14,15^. Given its low uncertainties, feasible transferability, and intrinsic relationship with water quality, FUI was recently promoted as a comprehensive water quality index for marine and inland waters, especially in large regions and over long time spans^7,12,14^.

In the past century, land-use changes, increasing urbanisation and industrialisation, population growth, together with apparent climate change have inevitably brought about changes in aquatic systems worldwide^16^. However, there is a lack of systematic water quality products or datasets available for global inland waters. Here, we present a time series dataset of FUI for large global lakes (including lakes and reservoirs, termed as ‘lake’ or ‘lakes’ for briefness hereafter) from 2000–2018 based on Moderate Resolution Imaging Spectroradiometer (MODIS) data. This dataset has a high value for providing unique information for the spatial patterns and long-term change trends of water colour worldwide over the past 19 years. These data could also be used in analyses in addressing scientific issues such as how water colour associated with hydrological parameters, climate change, and local anthropogenic activities at global and regional scales.

## Methods

### Water-leaving reflectance correction

The MODIS surface reflectance level-3 product (MOD09A1) was acquired from the Goddard Space Flight Center (GSFC) of the National Aeronautics and Space Administration (NASA) (http://ladsweb.nascom.nasa.gov/index.html). This global coverage product is 8-day composited data with 500 m spatial resolution that have been previously applied to inland water quality monitoring^7,17^. MOD09 has already been corrected for aerosol effect, Rayleigh scattering, and cirrus clouds and provides an estimation of surface reflectance. We performed a further water-leaving reflectance correction based on the minimum band value in the near infrared (NIR) to short wave infrared (SWIR) bands to remove the skylight reflection, residual aerosol effect, and sun glint for improved estimation of water-leaving reflectance (*R*_*rs*_)^18^. This correction method can be operationally applied to various types of inland waters over large areas with relatively stable and satisfactory performance^7,18^.

### Lake water body extraction and identification

**Fig. 1:**
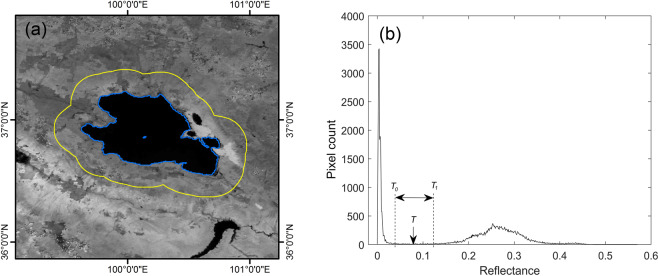
Diagram of the modified histogram bimodal method^19^. (**a**) The reflectance image of the 1640 nm band from MOD09A1 data; blue line denotes lake shoreline and yellow line denotes buffer boundary. (**b**) Histogram of the 1640 nm band for the whole expanded area enclosed by the buffer boundary including the water area and the buffer area, where T denotes the threshold value that is found as the valley value within the threshold range: here, T_0_ (0.04) and T_1_ (0.12) denote the left and right edges of the threshold range, respectively.

We used a modified histogram bimodal method to extract large inland water areas (>25 km^2^) automatically based on the reflectance of the 1640 nm band^7,19^. This band was selected because of the obvious reflectance difference between water and other land covers in the SWIR band. First, an initial rough water area was obtained based on the MOD09A1 Quality Assurance (QA) dataset where inland water pixels were marked. Then, during automatic selection of the threshold value (Fig. 1), a buffer zone was created around each connected water area with an area 1.5 times the initial water area. Based upon the expanded area including the initial water area and the buffer zone, a histogram of the 1640 nm reflectance was produced for the whole expanded area where water and other land-cover types would be distributed separately in the histogram within the two modes (Fig. 1(b)). Finally, the threshold value for this water was recognised as the valley value within a specific threshold range in the histogram (denoted as the range between T_0_ and T_1_ in Fig. 1). In this way, every water body found in the imagery could be identified separately with a threshold adapted to the water reflectance and its surrounding land-cover features, which can avoid misidentifications caused by one harmonised threshold value for all waters. In addition, clouds, cloud shadows, snow/ice, mixed pixels, and other noise pixels were identified using the MOD09A1 QA dataset, then removed before further analysis.

Following water body extraction from MODIS imagery, a normal water mask for lakes would be obtained where the occurrence frequency of the water pixels exceeds 30% during 2000–2018. The normal water mask represented the lake’s normal water area acquired by MODIS during 2000–2018 and served as the boundary in the following FUI statistical calculations. This removed the ephemeral water areas or low coverage water bodies from our dataset. Hence, the surface areas provided in this dataset are based on MODIS observations which may be a little different with the surface areas in other databases but indicated the valid water area calculated in this dataset. Besides, each lake’s centroid point was identified using the normal mask, and its latitude and longitude were then extracted. The geographical coordinates were used to identify a specified water body.

### FUI retrieval

We used a FUI retrieval algorithm for visible MODIS bands defined in previous research^7,14^, as summarized below:

1. CIE tristimulus X, Y, Z were calculated from the R, G, B bands of MOD09A1 after water-leaving correction using an RGB conversion method^6,20^:
  1 $$\begin{array}{l}X=2.7689R+1.7517G+1.1302B\\ Y=1.00R+4.5907G+0.0601B\\ Z=0.00R+0.0565G+5.5943B\end{array}$$
2. The chromaticity coordinates x, y were calculated by normalising X, Y, Z between 0 and 1^20^:
  2 $$\begin{array}{l}x=\frac{X}{X+Y+Z}\\ y=\frac{Y}{X+Y+Z}\end{array}$$
3. Hue angle α can be derived with x, y^9,10^:
  3 $$\alpha =\left(\arctan \left({\rm{y}}-\frac{1}{3},x-\frac{1}{3}\right){\rm{modulus}}\,2\pi \right)\times 180/\pi $$
  here, the hue angle α is in degrees and changes from 0° to 360° anti-clockwise starting from the positive x-axis at y - 1/3 = 0 in the CIE chromaticity diagram. We note that our previous publications^7,12,14^ have used a different definition for hue angle α (termed as α’ hereafter) where it increases in a clockwise direction starting from the negative axis at x - 1/3 = 0. This calculates the value of α’ using the formula *arctan2(x - 1/3, y - 1/3) + π* and α’ increases with FUI. However, this would not affect the FUI result because the same chromaticity coordinates of the 21 FUI colours^21^ were used to generate the FUI look-up table (Table 1).

**Table 1:** Chromaticity coordinates^21^ and corresponding hue angle α of FUI indices from 1 to 21.

FUI	x	y	α	α’
1	0.1914	0.1669	229.5330	40.4670
2	0.1990	0.1999	224.8037	45.1963
3	0.2100	0.2399	217.1473	52.8527
4	0.2265	0.2883	202.8305	67.1695
5	0.2459	0.3353	178.7020	91.2980
6	0.2662	0.3762	147.4148	122.5852
7	0.2908	0.4115	118.5208	151.4792
8	0.3154	0.4400	99.5371	170.4629
9	0.3367	0.4617	88.5017	181.4983
10	0.3633	0.4764	78.1648	191.8352
11	0.3862	0.4866	70.9617	199.0383
12	0.4024	0.4811	64.9378	205.0622
13	0.4162	0.4737	59.4234	210.5766
14	0.4313	0.4655	53.4431	216.5569
15	0.4457	0.4576	47.8847	222.1153
16	0.4606	0.4494	42.3707	227.6293
17	0.4753	0.4410	37.1698	232.8302
18	0.4887	0.4328	32.6477	237.3523
19	0.5033	0.4246	28.2408	241.7592
20	0.5155	0.4161	24.4487	245.5513
21	0.5283	0.4083	21.0471	248.9529

α’ denotes the differently defined hue angle used in our previous publications^7,12,14^.
4. To eliminate the colour difference caused by the MODIS visible band setting, we conducted a deviation delta (Δ) correction by modelling the *α* differences between human-eye-sensed true colour and MODIS-derived colour, following the idea proposed in previous research^9^. However, because we use different method to derive CIE tristimulus X, Y, Z (as shown in Eq. (1)), our correction equation is different with that in [9]:
  4 $${\rm{\bigtriangleup }}=-1.8185\times {\left(\frac{\alpha }{100}\right)}^{5}+87.01\times {\left(\frac{\alpha }{100}\right)}^{4}-486.65\times {\left(\frac{\alpha }{100}\right)}^{3}+1004.93\times {\left(\frac{\alpha }{100}\right)}^{2}-844.55\times \left(\frac{\alpha }{100}\right)+220.28$$
  5 $${\alpha }_{corrected}=\alpha +\Delta $$
5. Finally, the FUI of each pixel in the image was calculated from the corrected hue angle α using a look-up table (Table 1), which is determined through measuring the chromatic properties of Forel-Ule colour scale^21^.

### Monthly and yearly FUI calculation

All FUI images were produced using the 8-day composited MOD09A1 data from February 2000 to December 2018, and monthly FUI images were produced by removing outlier data in the time domain and averaging the remaining values in the same pixel location for one month. The time domain outlier data were recognised when outside the ‘μ ± 3σ’ window (μ denotes the average value and σ denotes the standard deviation). The monthly average FUI values were then calculated for each water body when the detected water pixels for one water body were >30% of those in the normal water mask to ensure the representativeness of the calculation. In the monthly average FUI calculation, outlier data in the spatial domain were removed and the remaining pixel values within the extent of the water body (identified by the normal water mask) were averaged. The spatial domain outlier data were recognised when outside the ‘μ ± 1.5σ’ window in order to avoid uncertainties caused by thin clouds, aerosol perturbations, or other noise, thereby ensuring more accurate monthly averaged FUI values for water bodies. Because lakes may be covered by clouds or other noise at times, there would be missing data for some lakes. To ensure the reliability of this time series dataset, lakes with less than six valid monthly data in one year from 2000–2018 were not included in this dataset. Because some Northern Hemisphere lakes may be covered or partly covered by ice during winter, their monthly FUI data were only calculated from boreal May to October each year. These frozen lakes were identified based on monthly climatological lake surface water temperature data provided by the ARC-Lake v3.0 dataset (http://www.laketemp.net/home_ARCLake/data_access.php)^22^. These lakes were only included if they had at least three valid monthly data points in each year. Finally, the missing data for lakes were filled through linear interpolation^23,24^. The yearly average FUI values were calculated for each water body by averaging the corresponding monthly average FUI.

## Data Records

The long-term FUI time series data are available via Figshare^25^.

General information for the 1049 investigated large lakes (>25 km^2^) around the world is compiled in ‘lake_info.csv’, where each row represents one lake and the columns are as follows:

1. Lake_id: Identifies each lake with MODIS tile and location number.
2. Lake_name: Lake name acquired from Google Earth and some lake database. A small part of lakes have blank names since we cannot find their names.
3. Lon: Longitudinal coordinate of the lake’s centroid point.
4. Lat: Latitudinal coordinate of the lake’s centroid point.
5. Lake_area: Surface area (km^2^) of the lake derived from the MODIS normal water mask.
6. Freezing: ‘Yes’ means the lake may freeze in winter, while ‘No’ means it would not freeze in winter.
7. Country/Region: Country or region in which the lake is located; international lakes are assigned to the country or region containing the centroid point and may be arbitrary for centroid points falling on the boundaries.
8. Continent: Continent in which the lake is located; international lakes may be arbitrarily assigned to one continent.

**Fig. 2:**
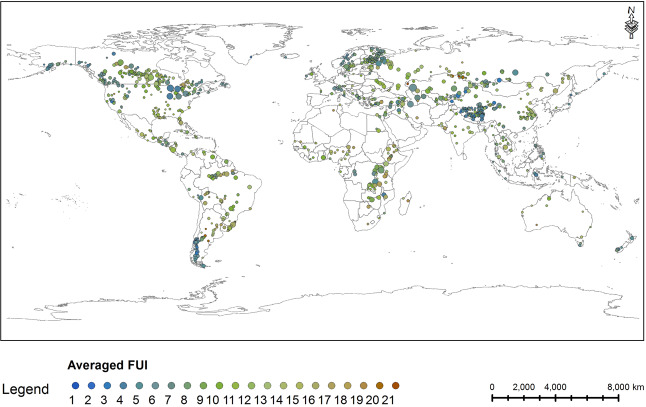
Average FUI of lakes from 2000–2018 in this dataset. Circle size is proportional to the lake surface area.

**Fig. 3:**
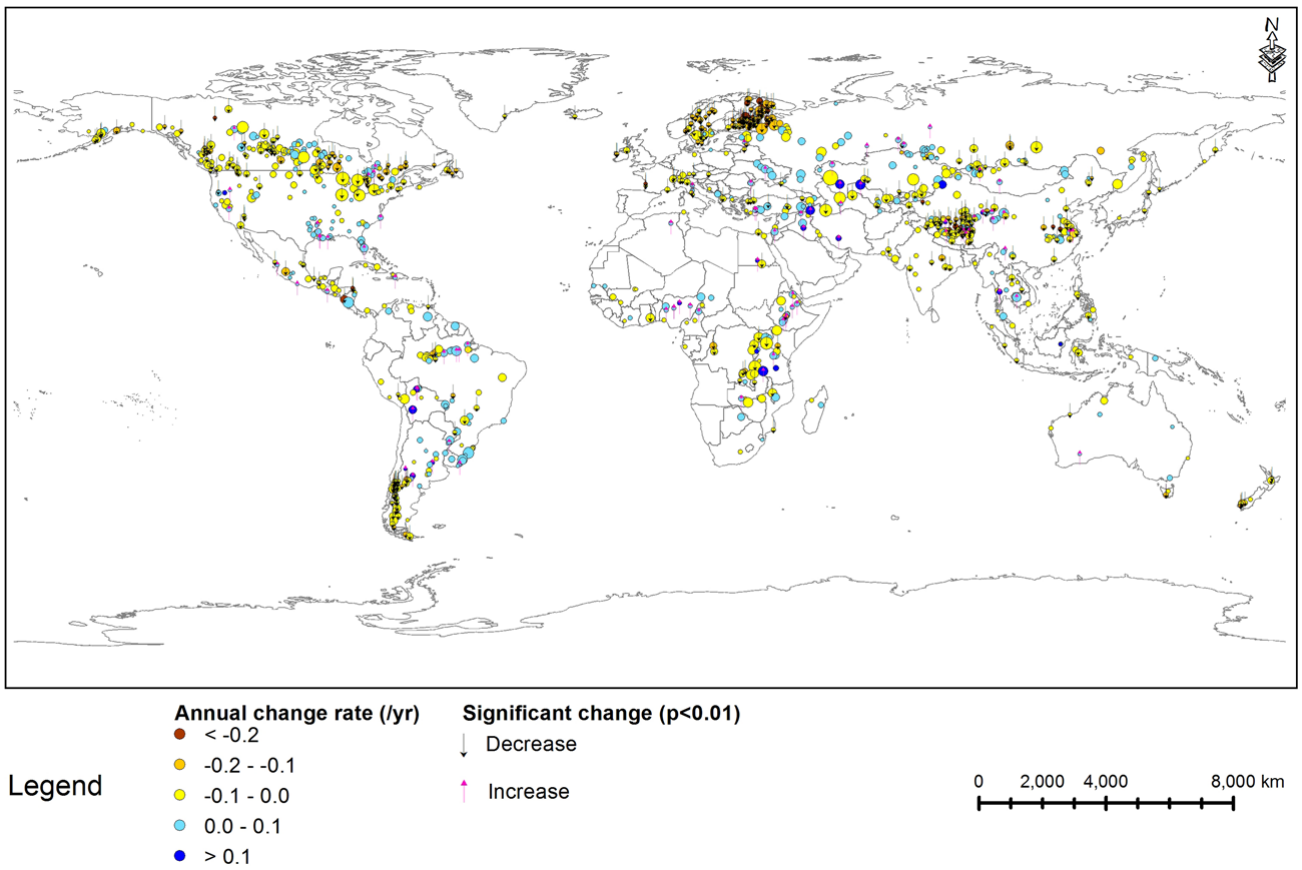
Annual change rate of FUI from 2000–2018 over lakes in this dataset. Circle size is proportional to the lake surface area.

The long-term monthly FUI data from February 2000 to December 2018 for lakes are compiled in the ‘monthly_FUI’ folder, in which the raw monthly FUI and filled monthly FUI data are provided in the ‘raw_monthly_FUI’ and ‘filled_monthly_FUI’ files, respectively. Monthly FUI data for freezing lakes are only provided from May to October for every year because ice cover changes the observed colour. Long-term yearly FUI data from 2000–2018 are compiled in the ‘yearly_FUI’ folder, in which the yearly mean FUI is provided in the ‘yearly_FUI.csv’ file. Average FUI of lakes from 2000–2018 is mapped in Fig. 2, and annual change rates are graphed in Fig. 3. Lakes have significant positive or negative yearly change trend (p < 0.01) in the nineteen years are also marked in Fig. 3.

## Technical Validation

### Quality control and assurance of the dataset

Quality control methods were embedded and executed during the processes of water-leaving reflectance correction, water body extraction, FUI image retrieval, and monthly- and yearly-average FUI calculation. After water-leaving reflectance correction and water body extraction were performed, clouds, cloud shadow, snow/ice, and other noise over the water area were identified using the QA flags attached to the MOD09A1 data and removed for further processing. The land adjacency effect was avoided by eroding the water areas with a 500 m distance^26^. To avoid data contamination by water bottom appearance, optically shallow water was excluded using a blue-band thresholding method assisted by visual interpretation using Google Earth images^7^. During monthly FUI image calculation, outliers at the pixel level were checked using the ‘μ ± 3σ’ criterion. During the summer-average FUI value calculation for each lake, water areas <30% of the normal surface area were removed to avoid average value biases caused by spatial variability in lakes. To avoid artificial errors, a set of scripts in the IDL programming language were composed for water extraction, FUI retrieval, and summer-average FUI calculation.

To assemble the summer-average FUI data for each lake, the lake ID was attached to each lake according to its centroid location, then the assembled dataset was cross-checked using a series of graphs and maps, allowing the identification of outliers and abnormal trends. We also compared the FUI change rates with other related water quality studies to confirm the results^27,28^.

### Validation with *in situ* data

Previous studies have shown that FUI can be derived from multispectral satellite data with high accuracy given its tolerance of aerosol perturbation and unfavourable viewing conditions, and the uncertainties in satellite water-leaving reflectance can be reduced during conversion to FUI^7,10^. We evaluated the FUI derived from MOD09 by comparison with *in situ* spectral data measured in our previous study^7^, which showed the uncertainties contained in the MODIS FUI data were <10%.

We further validated the MODIS FUI results using concurrent *in situ R*_*rs*_(*λ*) data, mainly from Chinese lakes. That is to say, the MODIS FUI is validated to the color of water itself without having any Secchi disk submerged, while there would be a Secchi disk put in the water during the traditionally FUI measurement using the handheld Forel-Ule water colour scale^29^. We note that there could be systematic biases between the satellite FUI data and the *in situ* FUI obtained using the handheld water colour scale assisted by a Secchi disk, but this is not the case in this study^30,31^. Here, the mean relative difference (MRD) and root mean square error (RMSE) were used to depict the uncertainties:

6 $${\boldsymbol{MRD}}=\frac{1}{{\boldsymbol{n}}}\mathop{\sum }\limits_{{\boldsymbol{i}}=1}^{{\boldsymbol{n}}}\frac{\left|{{\boldsymbol{x}}}_{{\boldsymbol{est}},{\boldsymbol{i}}}{{\boldsymbol{-x}}}_{{\boldsymbol{mea,i}}}\right|}{{{\boldsymbol{x}}}_{{\boldsymbol{mea}},{\boldsymbol{i}}}}\ast 100{\boldsymbol{ \% }}$$

7 $${\boldsymbol{RMSE\; =}}\sqrt{\frac{{\sum }_{{\boldsymbol{i}}=1}^{{\boldsymbol{n}}}{\left({{\boldsymbol{x}}}_{{\boldsymbol{est,i}}}{{\boldsymbol{-x}}}_{{\boldsymbol{mea,i}}}\right)}^{2}}{{\boldsymbol{n}}}}$$

where *x*_*est*_ denotes the estimated value, *x*_*mea*_ denotes the measured value, and *n* is the number of measurements.

**Fig. 4:**
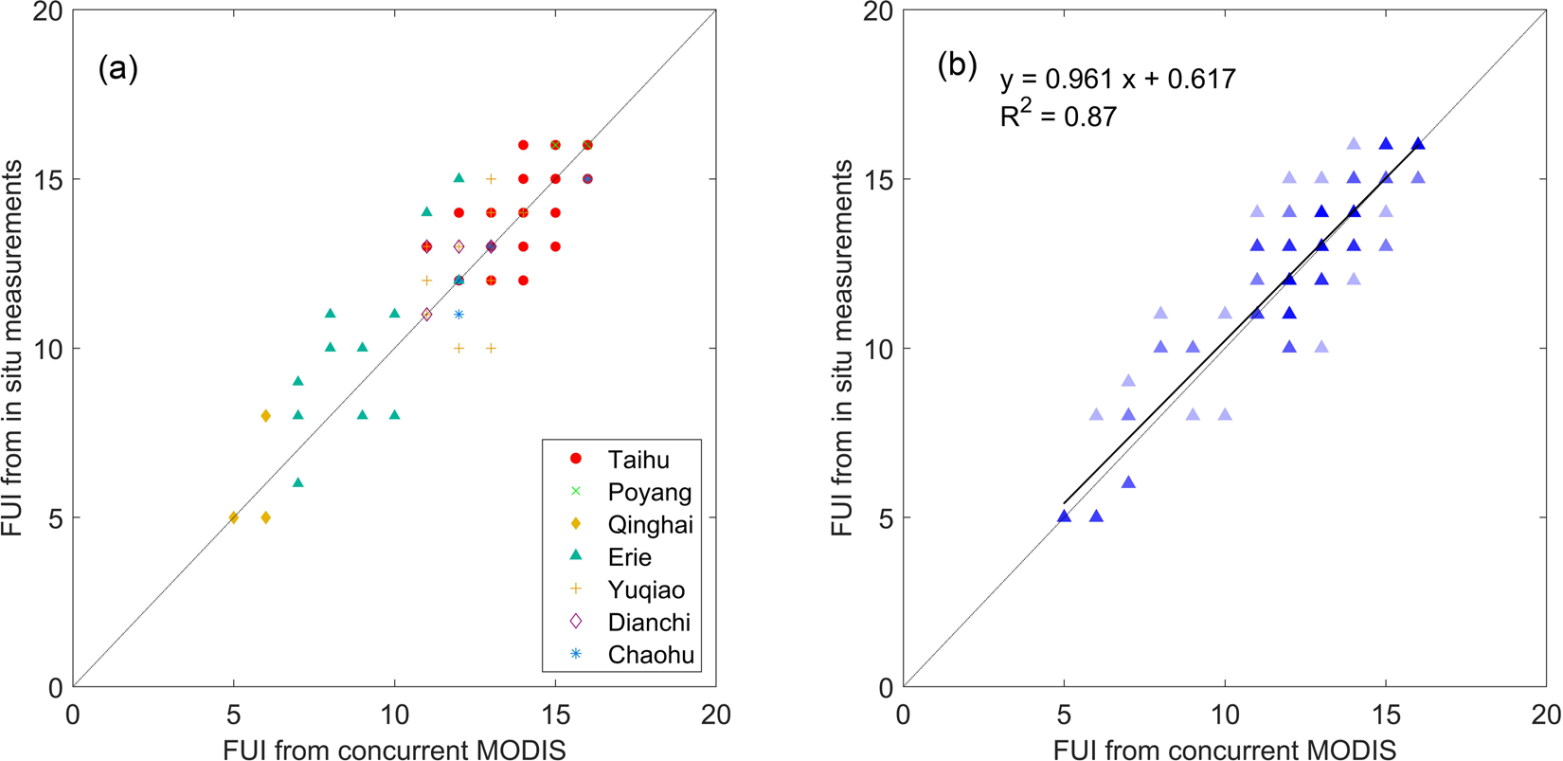
Scatterplots of the *in situ R*_*rs*_(*λ*) derived FUI and concurrent MODIS-derived FUI (**a**) by lake and (**b**) by 50% transparency (darker shading indicates higher data density).

The *in situ R*_*rs*_(*λ*) measurements were carried out in six large Chinese lakes with diverse water types ranging from clear and oligotrophic to turbid and eutrophic. In addition, *in situ R*_*rs*_(*λ*) data collected in Lake Erie (North America) were acquired from the SeaWiFS Bio-optical Archive and Storage System (SeaBASS) database and used to fill a gap in our data for moderately clear water (FUI ranging from 7–10). In the *in situ* measurements, above-water radiance measurements were conducted to derive water-leaving reflectance spectra for the sampling sites, then the water-leaving reflectance spectra were resampled to the MODIS bands and the FUI values were calculated. In the built of match-ups, the nearest pixel to the sampling location was selected in the MOD09 daily data (MOD09GA) and the time window was within 1 day. Finally, there are a total of 151 concurrent matchups in the seven lakes (Table 2). As shown in Fig. 4, the MRD between the MODIS FUI and *in situ* derived FUI was 6.5%, and the RMSE between them was 1.09. Given that the acceptable error level in satellite water colour products is ~30%^32,33^, our error rate of <10% demonstrates the validity of the MODIS FUI results. Moreover, the MODIS FUI was derived with a consistent methodology and dataset, further guaranteeing its performance for water colour change detection.

**Table 2:** Lake name, location, sampling date, and the number of match-ups (N) in the *in situ* dataset.

Lake name	Latitude	Longitude	Sampling date (YYYYMM)	N
Lake Taihu	31.1N	120.2E	200607, 200610, 200612, 200704	73
Lake Poyang	29.1N	116.3E	200910	4
Lake Chaohu	31.5N	117.5E	200906	5
Lake Dianchi	24.8N	102.7E	200912	4
Lake Qinghai	36.8N	100.3E	201408	10
Reservoir Yuqiao	40.0N	117.5E	201309, 201410	38
Lake Erie	41.6N	82.5W	201408	17

### Cross validation with diversity II data

**Fig. 5:**
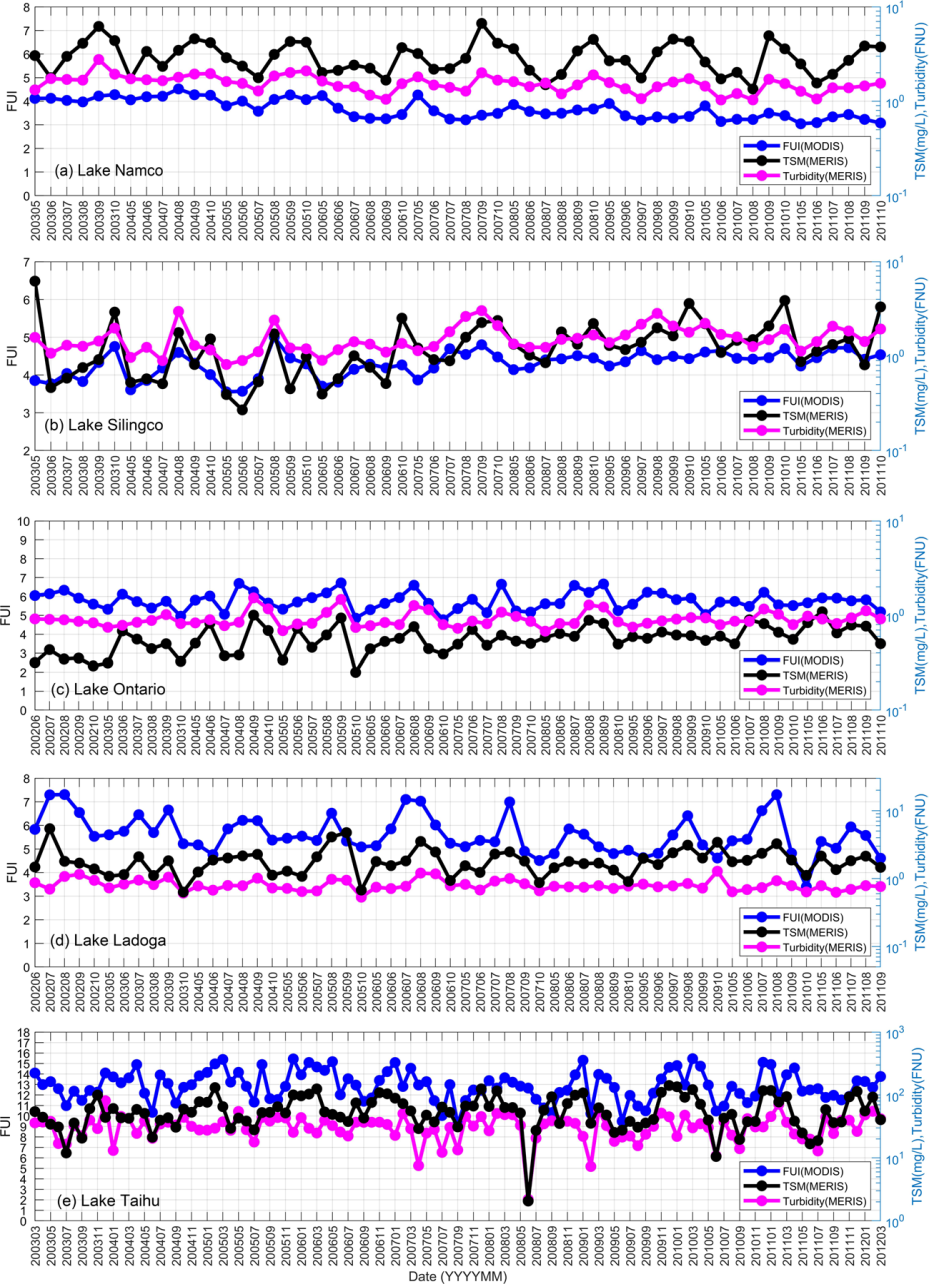
Comparison of FUI time series data derived from MODIS, and TSM and turbidity time series data derived from MERIS, in five representative lakes: (**a**) Lake Namco, (**b**) Lake Silingco, (**c**) Lake Ontario, (**d**) Lake Ladoga, and (**e**) Lake Taihu.

Water quality parameters (TSM and turbidity) provided by Diversity II dataset were used to cross validate the MODIS FUI dataset presented here in several large lakes around the world. The Diversity II datasets were produced from Medium Resolution Imaging Spectrometer (MERIS) data using optimised water quality retrieval algorithms for inland waters^34^. This dataset provides water quality data (e.g. Chl-a, TSM, and turbidity) for ~300 large lakes around the world from 2002–2012. As studies have shown^6,12,14,15^, FUI of water is well-correlated with Secchi disk depth and turbidity and can also indicate TSM in turbid waters with high suspended sediment. Therefore, our long-term monthly FUI data were compared with Turbidity and TSM monthly data from Diversity II dataset in several lakes including Lake Namco, Lake Silingco, Lake Ontario, Lake Ladoga and Lake Taihu (Fig. 5). This comparison showed that similar temporal patterns and trends in MODIS FUI and MERIS turbidity generally occurred in Lakes Namco, Silingco, and Ontario, which are relatively clear waters located in the Qinghai-Tibet Plateau and North America, respectively. In these lakes, MERIS TSM data basically had similar long-term trends with MERIS turbidity and MODIS FUI, but with some details that may differ. That is probably because water constituents other than TSM (such as CDOM) may also affect water colour and FUI. However, in Lake Taihu in eastern China, which is very turbid and dominated by TSM^35^, FUI and TSM basically had a better correlation than FUI and turbidity. In Lake Ladoga in northwestern Russia, the correlation between FUI and TSM is slightly higher than the correlation between FUI and turbidity, which suggest TSM have a little larger effect on FUI in this lake.

**Fig. 6:**
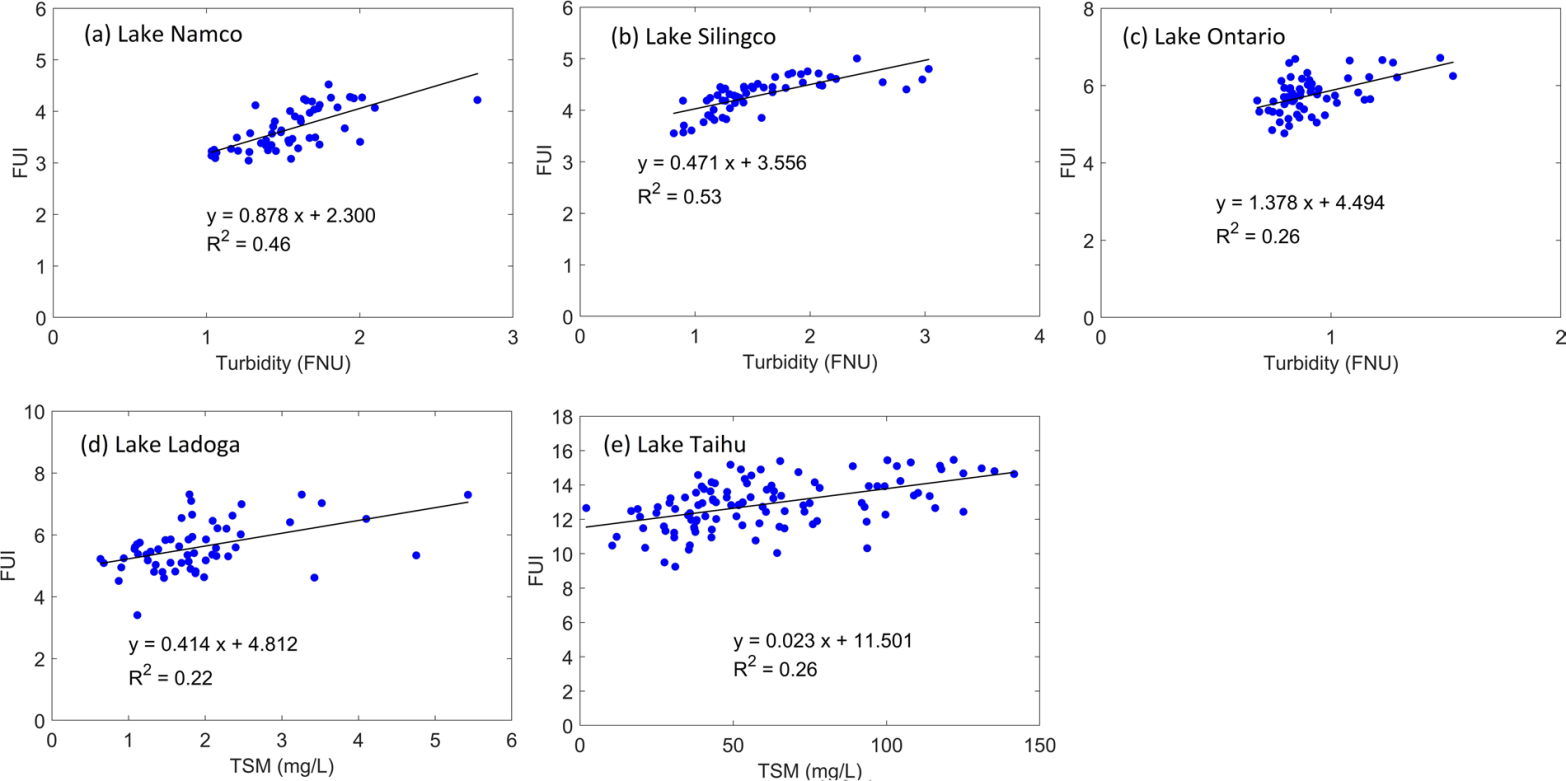
Scatterplots of MERIS-derived turbidity or TSM and MODIS-derived FUI in five representative lakes: (**a**) Lake Namco, (**b**) Lake Silingco, (**c**) Lake Ontario, (**d**) Lake Ladoga, and (**e**) Lake Taihu.

Figure 6 shows correlations between the monthly FUI and turbidity in Lake Namco, Lake Silingco, and Lake Ontario, and the correlations between monthly FUI and TSM in Lake Ladoga and Lake Taihu. Our FUI data and Diversity II Turbidity or TSM data generally showed good agreement, with correlation coefficients (R) ranging from 0.47–0.73, consistent with previous research showing that FUI can be used as an indicator of water clarity^12,14,15^. The correlation coefficients in Lakes Namco and Silingco were higher than those in the other three lakes, which are reasonable because water constituents in the latter three are generally more complicated^28,35–38^. In addition to the suspended solids quantified by TSM and turbidity, CDOM may also play an important role in driving water colour changes in some saline lakes and lakes surrounded by forests or agriculture farmland^39^, so in these cases the correlation between FUI and turbidity and TSM might be weak. As the Diversity II data and our FUI data were produced using different satellite data (MERIS and MODIS, respectively), the good agreements between the two datasets shown here also demonstrate the reliability of both the two satellite data for use in studying long-term water colour and water quality parameters for inland waters when assisted by proper atmospheric corrections.

## Data Availability

The IDL code named MODIS_FUI.pro for calculating FUI from MOD09A1 data is also available via Figshare^25^. We note that the code contains a few steps that need ENVI software, so that it needs to be run under the ENVI + IDL environment. The ENVI version 5.3 and the IDL version 8.5 were used in the code development.

## References

[1] Williamson CE, Saros JE, Vincent WF, Smol JP (2009). Lakes and reservoirs as sentinels, integrators, and regulators of climate change. Limnol. Oceanogr..

[2] Hipsey MR (2019). A general lake model (GLM 3.0) for linking with high-frequency sensor data from the Global Lake Ecological Observatory. Network (GLEON). Geosci. Model Dev..

[3] Klein I, Gessner U, Dietz AJ, Kuenzer C (2017). Global waterpack – a 250, m resolution dataset revealing the daily dynamics of global inland water bodies. Remote Sens. Environ..

[4] Palmer SCJ, Kutser T, Hunter PD (2015). Remote sensing of inland waters: challenges, progress and future directions. Remote Sens. Environ..

[5] Malthus, T. J., Hestir, E. L., Dekker, A. G. & Brando, V. E. The case for a global inland water quality product. *2012 IEEE Int. Geosci. Remote Sens. Symp*. (2012).

[6] Wang S, Li J, Shen Q, Zhang B, Zhang F, Lu Z (2015). MODIS-based radiometric color extraction and classification of inland water with the Forel-Ule scale: a case study of Lake Taihu. IEEE J. Select. Topics Appl. Earth Obs. Remote Sens..

[7] Wang S (2018). Trophic state assessment of global inland waters using a MODIS-derived Forel-Ule index. Remote Sens. Environ..

[8] Wernand MR, Hommersom A, van der Woerd HJ (2013). MERIS-based ocean colour classification with the discrete Forel–Ule scale. Ocean Sci..

[9] Van der Woerd HJ, Wernand MR (2015). True colour classification of natural waters with medium-spectral resolution satellites: SeaWiFS, MODIS, MERIS and OLCI. Sens..

[10] Van der Woerd HJ, Wernand MR (2018). Hue-angle product for low to medium spatial resolution optical satellite sensors. Remote Sens..

[11] Wernand, M. R., van der Woerd, H. J. & Gieskes, W. W. C. Trends in ocean colour and chlorophyll concentration from 1889 to 2000, worldwide. *PLOS ONE* 8 (2013a). 10.1371/journal.pone.0063766 PMC3680421 23776435

[12] Wang S (2020). Changes of water clarity in large lakes and reservoirs across China observed from long-term MODIS. Remote Sens. Environ..

[13] Pitarch J, van der Woerd HJ, Brewin RJW, Zielinski O (2019). Optical properties of Forel-Ule water types deduced from 15 years of global satellite ocean color observations. Remote Sens. Environ..

[14] Li J (2016). MODIS observations of water color of the largest 10 lakes in China between 2000 and 2012. Int. J. Digit. Earth.

[15] Garaba S, Friedrichs A, Voß D, Zielinski O (2015). Classifying natural waters with the Forel-Ule colour index system: results, applications, correlations and crowdsourcing. Int. J. Environ. Res. Public Health.

[16] Tang Q (2019). Global change hydrology: terrestrial water cycle and global change. Sci. China Earth Sci..

[17] Feng L, Hu C, Li J (2018). Can MODIS land reflectance products be used for estuarine and inland waters?. Water Resour. Res..

[18] Wang S (2016). A simple correction method for the MODIS surface reflectance product over typical inland waters in China. Int. J. Remote Sens..

[19] Zhang F (2018). A simple automated dynamic threshold extraction method for the classification of large water bodies from Landsat-8 OLI water index images. Int. J. Remote Sens..

[20] CIE. *Commission Internationale de l’Eclairage Proceedings 1931* (Cambridge Univ. Press, 1932).

[21] Novoa, S., Wernand, M. R. & van der Woerd, H. J. The Forel-Ule scale revisited spectrally: preparation protocol, transmission measurements and chromaticity. *J. Europ. Opt. Soc. Rapid Publ*. **8** (2013).

[22] MacCallum SN, Merchant CJ (2012). Surface water temperature observations of large lakes by optimal estimation. Can. J. Remote Sens..

[23] Verger A, Baret F, Weiss M (2011). A multisensor fusion approach to improve LAI time series. Remote Sens. Environ..

[24] Kandasamy S, Baret F, Verger A, Neveux P, Weiss M (2013). A comparison of methods for smoothing and gap filling time series of remote sensing observations-application to MODIS LAI products. Biogeosciences.

[25] Wang S (2020). figshare.

[26] Feng L, Hu C (2017). Land adjacency effects on MODIS Aqua top‐of‐atmosphere radiance in the shortwave infrared: Statistical assessment and correction. J. Geophys. Res. Oceans.

[27] Feng L, Liu J, Ali TA, Li J, Li J, Kuang X (2019). Impacts of the decreased freeze-up period on primary production in Qinghai Lake. Int. J. Appl. Earth Obs..

[28] Liu C (2017). Remote sensing-based estimation of lake water clarity on the Tibetan Plateau. Prog. Geogr..

[29] Wernand, M. R. & van der Woerd, H. J. Spectral analysis of the Forel-Ule Ocean colour comparator scale. *J. Eur. Opt. Soc.-Rapid*, **5** (2010).

[30] Pitarch J (2017). Biases in ocean color over a Secchi disk. Opt. Express.

[31] Nie, Y., Guo, J., Sun, B., & Lv, X. An evaluation of apparent color of seawater based on the *in-situ* and satellite-derived Forel-Ule color scale. *Estuar., Coast. Shelf S*., **107032** (2020).

[32] Mouw CB (2015). Aquatic color radiometry remote sensing of coastal and inland waters: challenges and recommendations for future satellite missions. Remote Sens. Environ..

[33] IOCCG. *Earth Observations in Support of Global Water Quality Monitoring*. Greb, S., Dekker, A. & Binding, C. (eds.) (IOCCG Report Series, No. 17, International Ocean Colour Coordinating Group, Dartmouth, Canada, 2018).

[34] Odermatt D, Danne O, Philipson P, Brockmann C (2018). Diversity II water quality parameters from ENVISAT (2002-2012): a new global information source for lakes. Earth Syst. Sci. Data.

[35] Shi K, Zhang Y, Liu X, Wang M, Qin B (2014). Remote sensing of diffuse attenuation coefficient of photosynthetically active radiation in Lake Taihu using MERIS data. Remote Sens. Environ..

[36] Wang, J. et al. Investigation of bathymetry and water quality of Lake Nam Co, the largest lake on the central Tibetan Plateau, China. *Int. Symp. Tibetan Plateau Himalaya-Karakorum-Tibet Workshop* (2009).

[37] Naumenko MA (2008). Seasonality and trends in the Secchi disk transparency of Lake Ladoga. Hydrobiologia.

[38] Howell ET (2018). Influences on water quality and abundance of cladophora, a shore-fouling green algae, over urban shoreline in Lake Ontario. Water.

[39] Wen Z (2020). A national-scale data set for dissolved carbon and its spatial pattern in lakes and reservoirs across China. Sci. Data.

